# Retraction: Assessment and Evaluation of Social Engagement in Dermatology Residency Programs on Instagram: Cross-sectional Study

**DOI:** 10.2196/106666

**Published:** 2026-08-19

**Authors:** JMIR Publications Editorial Office

**Affiliations:** 1 JMIR Publications Toronto, ON

The JMIR Publications Editorial Office is retracting the article “Assessment and Evaluation of Social Engagement in Dermatology Residency Programs on Instagram: Cross-sectional Study” [1] due to concerns regarding the integrity of the peer review process.

Author Chapman Wei responded, stating that there were no conflicts of interest with the reviewers. However, the Editorial Office has determined that there is sufficient evidence indicating that the peer review process was compromised to proceed with a retraction.

Chapman Wei and Nagasai Adusumilli responded but did not explicitly state whether they agreed or disagreed with the decision to retract the article. The remaining authors were contacted but did not respond to communications.
